# Preparing medical students by simulating on‐call shifts

**DOI:** 10.1111/medu.15663

**Published:** 2025-03-10

**Authors:** Amber Ahmed‐Issap, Jessica Bialan, Michael Eastwood, John Barnes

## 1 WHAT PROBLEMS WERE ADDRESSED?

Final year medical students possess a wealth of knowledge but lack experience and confidence putting theory into clinical practice. This impacts readiness for on‐call duties during training for new doctors.^1^ Simulation of on‐call duties is a novel approach to improve clinical preparedness.^1^ However, we are not aware of programmes being able to deliver this for large cohorts.

## 2 WHAT WAS TRIED?

We conducted a 3‐hour on‐call simulation for final year medical students. Seven students participated per session, three sessions occurred per day, totalling 21 students. The entire year group of 95 students participated in five on‐call simulation days. Eight facilitators ran seven clinical stations simultaneously, each with a facilitator recording individual student feedback. A lead tutor acted as a senior registrar whom students called for advice. Paid performers or faculty acted as simulated patients (SPs). Students were provided with an electronic pager for authentic communication. Each facilitator paged students to their station according to a timetable. Facilitators recorded the time paged, return of pager call and arrival time to assess students' time management. The duration between pages varied with some in quick succession to replicate real‐life. Students were required to clinically prioritise tasks.

We offer the following detail for those wishing to run an on‐call simulation: Station 1 focused on common prescribing tasks, e.g. managing hyperkalaemia. Station 2 required students to call a senior clinician, e.g. to protocol a CT scan or obtain management advice. Stations 3 and 5 involved reviewing a patient e.g. to assess a red swollen leg or death verification. Station 4 involved a difficult discussion with a SP, e.g. discussing a cancelled surgery. ‘The Mess’ room allowed students to rest and organise themselves with the lead tutor present for pastoral support.

At 40 minutes remaining, students were paged for ‘handover’ to practice communicating jobs to another doctor. The simulation completed with a debrief session to share general feedback. Students completed an anonymous survey to assess satisfaction and pitfalls.

## 3 WHAT LESSONS WERE LEARNED?

Our simulation successfully introduced students to the challenges of on‐call shifts. Student and faculty feedback was positive and confirmed on‐call simulation improved clinical preparedness for several reasons. Learning how to receive, record and respond to pages, and cope with the volume, was considered highly beneficial. Moving between stations and floors according to pages reflected real‐life practical demands. Students appreciated ‘stepping‐up’ as a doctor by independently applying their clinical knowledge in common scenarios with senior phone support. Students practiced delivering and receiving referrals and prioritising tasks, with both valued learning outcomes.

Challenges included the design and execution requiring one‐to‐one faculty input and difficulties with student flow. Sending pages complicated station facilitation. Students backlogged on tasks meant others missed some stations. To preserve flow, we suggest setting a station time‐limit and allocating a ‘floating’ facilitator to takeover paging.

We demonstrate how on‐call simulation is feasible for whole year groups and potentially sustainable with increasing medical school cohorts. With the need for clinical preparedness on graduation, medical programmes should consider investing in on‐call simulation.

## AUTHOR CONTRIBUTIONS

**Amber Ahmed‐Issap:** Conceptualization; data curation; writing – original draft; writing – review and editing; methodology; formal analysis; investigation; visualization; project administration. **Jessica Bialan:** Data curation; writing – review and editing; methodology; visualization; project administration. **Michael Eastwood:** Methodology; writing – review and editing; visualization; project administration. **John Barnes:** Methodology; writing – review and editing; visualization; project administration.

## CONFLICTS OF INTEREST STATEMENT

All authors declare that there is no conflict of interest.

## ETHICS STATEMENT

This research did not require any ethical approval.

## Data Availability

The data that support the findings of this study are available from the corresponding author upon reasonable request.
